# Federated learning: a privacy-preserving approach to data-centric regulatory cooperation

**DOI:** 10.3389/fdsfr.2025.1579922

**Published:** 2025-05-26

**Authors:** Alexander Horst, Paul Loustalot, Sanjeev Yoganathan, Ting Li, Joshua Xu, Weida Tong, David Schneider, Nicolas Löffler-Perez, Erminio Di Renzo, Michael Renaudin

**Affiliations:** ^1^ Swissmedic 4.0 and Medical Device Vigilance, Swissmedic, Swiss Agency for Therapeutic Products, Bern, Switzerland; ^2^ Quinten Health, Paris, France; ^3^ Division of Medical Devices, The Danish Medicines Agency, Copenhagen, Denmark; ^4^ National Center for Toxicological Research, U.S. Food and Drug Administration, Jefferson, AR, United States

**Keywords:** federated learning, regulatory sciences, medical devices, risk assessment, swissmedic, data privacy

## Abstract

Regulatory agencies aim to ensure the safety and efficacy of medical products but often face legal and privacy concerns that hinder collaboration at the data level. In this paper, we propose federated learning as an innovative method to enhance data-centric collaboration among regulatory agencies by enabling collaborative training of machine learning models without the need for direct data sharing, thereby preserving privacy and overcoming legal hurdles. We illustrate how Swissmedic, the Swiss Agency for Therapeutic Products, together with its partner agencies, proposes to use federated learning to improve TRICIA, an AI tool for assessing incoming reports of serious incidents related to medical devices. This approach enables the development of robust, generalisable risk assessment models that can potentially improve current processes. A proof of concept was deployed and thoroughly tested during the 14th Global Summit on Regulatory Science using synthetic data with participants from Swissmedic, the U.S. Food and Drug Administration (FDA), and the Danish Medicines Agency (DKMA), with promising initial results. This innovation has the potential to serve as a roadmap for other regulators to adopt similar approaches to optimize their own regulatory processes, contributing to a more integrated and efficient regulatory environment worldwide.

## 1 Introduction

Regulatory science, encompassing the scientific disciplines and methodologies used to assess and ensure the safety, efficacy, and quality of medical products and devices, is essential for developing effective regulatory policies and procedures. Collaboration between regulatory authorities is critical to maintaining sound and effective oversight as these partnerships promote harmonisation of standards and practices. This enables a more coherent and comprehensive approach to the protection of public health.

At the interface of regulatory science and data science, various initiatives have been launched to explore innovative use cases and develop best practices across organisations. An example is the Large Language Model (LLM) Taskforce of the Global Coalition for Regulatory Science Research (GCRSR), which deals with the safe and efficient use of LLMs in regulatory science (LLMs, 2024). However, collaboration at the data level brings new challenges. In particular, the sharing of sensitive personal data such as patient information raises significant privacy issues and is therefore often considered unethical (Sprenkamp et al., 2023). Strict regulations such as the General Data Protection Regulation (GDPR) and the Health Insurance Portability and Accountability Act (HIPAA), which are intended to ensure the protection of sensitive personal data, simultaneously limit the possibilities for collaboration. Furthermore, additional confidentiality legislation, for example, relating to trade secrets, may also apply. Consequently, direct data sharing is frequently impossible.

Regulatory data can be shared using several privacy protection methods, each with its own implications. One of the most common is de-identification, which removes or obscures explicit personal identifiers such as names or ID numbers. A more stringent variant is anonymisation, which involves altering or generalizing data so that individuals are no longer identifiable, even when combined with other data sets. While these techniques preserve privacy, they can end up removing important context; for example, removing identifiers or fine-grained demographics can obscure important risk factors and make it harder for algorithms to identify useful patterns. Furthermore, data that has been treated as anonymised or de-identified may still pose a risk of re-identification if an adversary can link it to other data (Truong et al., 2021). In practice, true anonymity is difficult to achieve, and there is generally a trade-off between privacy and utility in such techniques.

Another approach is to share only aggregated information rather than individual-level information. For example, regulatory agencies may exchange summary statistics or trend reports rather than the underlying raw data. Aggregated information, by definition, reveals much less about a particular individual, which in turn protects privacy (Sprenkamp et al., 2023). A drawback of over-aggregation, however, is that it can mask subtle signals a model could detect by ignoring individual data points in favour of averages or sums. Synthetic data is an area that has recently been pursued by regulators as an effective option. Synthetic datasets are artificially created to reflect the statistical properties of real datasets, without including real personal data. Collaboration is made possible because real patient data is not revealed. However, synthetic data tend to lack the heterogeneity and complexity present in real data, causing models to miss critical subtleties or be biased (Synthetic Data, 2025). In general, the processes of de-identification/anonymisation, aggregation and data synthesis help to facilitate data sharing within privacy constraints, but each method has inherent drawbacks.

Regulatory authorities face the challenge of finding a balance between data utility and privacy to ensure effective regulatory co-operation. In this paper, we propose federated learning as a solution that maintains the decentralisation of data by sharing only aggregated insights, ensuring privacy protection from the outset. This approach enables a data-centric model of collaboration between regulatory authorities and improves cooperation without jeopardising data protection. Besides providing a theoretical description of the approach a proof of concept was developed during the 14th Global Summit on Regulatory Sciences organized by the Global Coalition for Regulatory Science Research (GCRSR).

## 2 A primer on federated learning

Federated learning (McMahan et al., 2016), introduced by Google in 2016, was developed in response to the increasing computing power of devices (Poushter, 2024) (Anderson, 2024) and the growing importance of data protection and data security (Xie et al., 2020), (Gong et al., 2020). This machine learning (ML) technology makes it possible to train models directly on distributed devices or servers without having to collect or transfer the data centrally. As the model learns locally from the data without it being sent to a central location, federated learning significantly improves data security and privacy protection. This approach is particularly valuable in sensitive areas such as healthcare, as it enables insights from multiple data sources while preserving individual privacy.

Federated learning has been used in several projects through the years. Google uses it for next-word prediction when typing on a keyboard so that the model learns from each individual user (Hard, 2019). It has also been used in healthcare, for example, to predict future oxygen requirements of patients infected with SARS-COV-2 during the COVID-19 pandemic (Flores et al., 2021). Finally, it has also be used in intergovernmental collaboration, for example, in the case of the Ukrainian refugee crisis (Sprenkamp et al., 2024). To our knowledge, no federated learning project has been implemented so far by regulatory authorities yet.

In the practical implementation of federated learning, several users, known as clients, are involved. Their communication with each other is generally orchestrated by a central server. Each client remains the owner of its data, which is not passed on to the central server or other clients. The main goal of federated learning is to optimise a common model, the so-called global model, through local training with the data of the individual clients. The central server receives the locally updated models from the clients and merges them into an improved global model.

**FIGURE 1:**
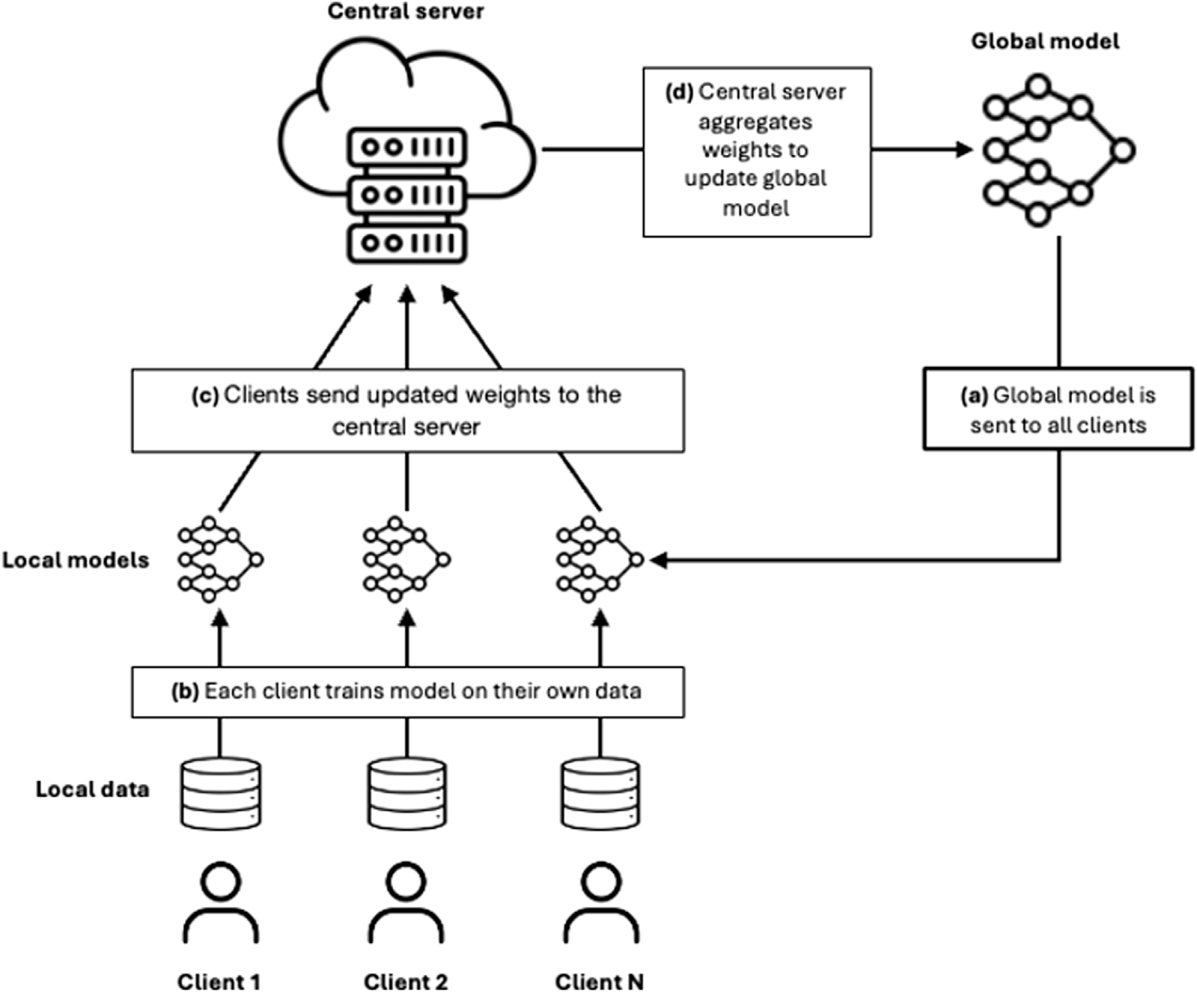
Schematic diagram of a federated learning process. This figure describes the main elements of the process (clients, central server, global model) and the four steps: **(a)** the global model is sent to all clients; **(b)** the clients train the model on their own data; **(c)** they send the updated weights to the central server; **(d)** central server aggregates the updated weights to create an updated global model.

Federated learning works through a process that takes place in rounds (Figure 1) and consists of several successive steps. First, the global model is distributed from the central server to the clients. In each round, either all or a subset of the clients can participate in the process. The participating clients train the model with their local data. After training, each client sends its updated model back to the central server. There, the different versions of the model weights are aggregated into an updated global model. This updated global model is then sent back to the clients so that they can evaluate it with their respective test datasets.

Model aggregation plays a significant role in updating the overall model in federated learning. In each communication round, information, usually model parameters, are aggregated from all participants. By aggregating only model parameters rather than raw data, privacy is effectively protected. The aggregation methods differ depending on the structure (Qi et al., 2024). In centralised federated learning, a central server collects and aggregates the models of the individual clients. In contrast, decentralised federated learning relies on peer-to-peer communication for model aggregation, making a central server superfluous. Research into different aggregation methods is driving the advancement of federated learning with the aim of optimising model performance, minimising communication overhead while addressing data security and privacy concerns (McMahan et al., 2016). In each aggregation, the weights of the clients are weighted proportionally to the number of data points used for training and then averaged with the weights of the other clients participating in the round.

Federated learning facilitates the development of models using data from different institutions without the need to centralise this data. This approach takes advantage of the growing amount of health data collected by different regions and organisations. By integrating diverse and heterogeneous datasets, federated learning aims to improve the generalisability and performance of models. In addition, federated learning enables continuous learning and updating of models in real time without the need for data transfer. This makes it particularly suitable for dynamic and constantly evolving health science datasets. This ensures that the best performing model is always available, while more traditional, static approaches could become outdated or obsolete over time.

## 3 A critical review from the perspective of a regulatory authority

The implementation of federated learning within a regulatory framework requires careful consideration of several important security, legal and ethical aspects. Regulatory authorities must ensure that the use of data and modelling of learning outcomes is traceable and that these processes comply with the GDPR and other relevant data protection laws. A key aspect is securing the communication channels, e.g., by using the Transport Layer Security (TLS) protocol to protect data transmission, including model weights and performance metrics (Sun et al., 2021), (Hidayat et al., 2023). In addition, legal and ethical accountability requires that modelling decisions and more generally, expert decisions based on the output of a ML model are transparent to all stakeholders and can be explained in a comprehensible manner which is particularly important in a regulatory context. Additionally, every decision of an AI model must be judicially reviewable, which means that the machine output must be transparent in the sense that it must be comprehensible to humans. Rapid technological progress also brings ethical and regulatory challenges, including global inequalities and privacy risks (Hassan et al., 2021). In addition to this, the newly introduced (European Union, 2024) may impact the requirements for implementing federated learning. While federated learning itself is not explicitly regulated by the AI Act, its use in AI systems is subject to the Act’s provisions. Depending on the specific implementation, an AI system utilizing federated learning could be classified as a high-risk product, necessitating strict adherence to protocols to ensure compliance with the Act. Although there is a potential for AI systems employing federated learning to be categorised as high-risk, this approach aligns well with the principles of the GDPR. Federated learning emphasizes privacy-by-design and secure data handling—both of which are reinforced by the requirements outlined in the AI Act. The most important aspects to consider when developing a federated learning use case in a regulatory context are explained below.

### 3.1 Data protection and confidentiality

Regulatory authorities work with highly sensitive information, including personal health data and trade secrets. As a result, the data pool of a typical regulatory authority is covered by both data protection and confidentiality legislations. Federated learning supports compliance with strict data protection and confidentiality regulations such as the General Data Protection Regulation (GDPR) in the European Union, the Health Insurance Portability and Accountability Act (HIPAA) in the United States, and the revised Data Protection Act (DSG) in Switzerland by storing sensitive health data on local servers or devices and transmitting only aggregated model updates (Truong et al., 2021). By training models locally and only aggregating updates, federated learning significantly reduces the risk of data breaches and unauthorised access. This enables effective collaboration between different healthcare providers, research organisations and regulatory authority without the need to share sensitive data directly which is a cornerstone to regulations such as the GDPR. However, these regulations require more than just ensuring data security by establishing and documenting a valid legal basis for processing personal data. Each of the participating agency must independently obtain an adequate legal basis, such as explicit consent, contractual necessity, legal necessity, protection of essential interests, or legitimate interest, before processing personal data (Nišević et al., 2022). The overarching federated research or joint endeavour must also ensure that it collectively complies with these legal requirements, thereby meeting the GDPR requirements holistically.

In addition, differential privacy is increasingly being used to improve privacy in federated learning systems. Common approaches include centralised, local and distributed differential privacy (Zhang et al., 2023). Local differential privacy is particularly popular as it provides strong privacy guarantees by adding noise to local model updates before aggregation (Sun et al., 2021). However, local differential privacy can have a significant impact on model accuracy. Recent work has focused on optimising federated learning techniques, such as adaptive Gaussian clipping, to strike a balance between privacy and accuracy (Hidayat et al., 2023).

### 3.2 Model security

Although federated learning improves privacy and confidentiality by preventing the direct sharing of data, the security of the globally shared model remains a challenge. Recent research has shown that it is in theory possible to extract potentially sensitive information from language models (He et al., 2022). Although this is currently a manual and tedious task and may only apply to certain model architectures, it emphasises the need for a secure model repository.

Besides theft of the global modal, a second major threat is model poisoning, where attackers could inject malicious data into the training process (Xia et al., 2023). Model poisoning can lead to compromised model accuracy and integrity, potentially embedding harmful biases or vulnerabilities into the final aggregated model.

The AI Act highlights the need for resilience against these types of adversarial attacks and threats. Since systems like federated learning can be susceptible to threats or attacks like inference attack, where the attacker could extract potentially sensitive information that is not directly included in the training data (IBM, 2025), techniques such as differential data protection or TLS via Multi Party Computation (MPC) are both required to minimise this risk, and comply with the regulation in order to ensure robustness and security. For instance, differential privacy prevents sensitive training data from being extracted from models by adding random mutations, often referred to as noise, to the training data. Studies have shown that a noise content of up to 30% in the training data can increase security, while the impact on model performance is moderate (Xia et al., 2023).

### 3.3 Decentralised and heterogeneous data

Additional challenges for federated learning arise from the volume and heterogeneity of the data. Effective model training requires large amounts of data, which remain decentralised in federated learning. This can lead to some clients not having enough data available, making it difficult to improve the model. In addition, the data in federated learning environments is often not independently and identically distributed. In healthcare, for example, patient data varies significantly from country to country and reflects different population characteristics, which further complicates model training. Furthermore, while regulatory processes within regulatory authorities may be similarly structured, the specific predictions they seek to make vary, requiring additional complexity in model fitting. For example, multiple regulatory authorities may be interested in performing a risk assessment on adverse event reports, but they may be interested in different risk factors. The first may be interested in predicting the severity of an event (patient harm), while the second agency may be interested in predicting the likelihood of similar events occurring. These differences in prediction goals mean that a single federated model must accommodate multiple goals or label definitions, adding complexity to model training.

### 3.4 Operational aspects relating to costs, intellectual property and data ownership

Regulatory authorities also need to address the issue of information asymmetry, especially in inadequately resourced environments, which may be at a disadvantage. To avoid conflicts in relation to the global models used in federated learning, intellectual property rights and ownership of the data need to be clearly defined (Sprenkamp et al., 2023). Another important aspect is to ensure interoperability between different systems and organisations, which is an essential prerequisite for safe and effective collaboration, especially when multiple regulatory authorities are involved.

In addition, the communication costs associated with federated learning pose a major challenge. In systems with millions of clients, the communication overhead for model updates can be significant, which can affect the efficiency and scalability of the process (Zhang et al., 2021).

### 3.5 Computational resources

As the volume of data in regulatory science continues to grow, so does the demand for the computational resources needed to perform advanced analyses (Zhao and Li, 2011). Traditional machine learning (ML) approaches that rely on centralised data pooling often require extensive computational infrastructure, including high-performance hardware such as multiple GPUs (Amaral et al., 2017). Federated learning offers a promising alternative by leveraging distributed computing resources across multiple agencies or stakeholders, allowing complex models to be trained without centralising data. This approach not only reduces the time and cost of the learning process, but also addresses critical privacy and security concerns by keeping data local to its owners. By optimising resource utilisation and decentralising data management, federated learning provides a scalable and cost-effective solution tailored to the unique challenges of regulatory science, fostering collaborative innovation while maintaining compliance with legal and ethical standards.

### 3.6 Performance evaluation

As mentioned earlier, different processes can result in not only different data structures, but also different performance metrics worth monitoring. These discrepancies affect both the ML metrics targeted during training and business-related metrics, such as the percentage of missed high-risk cases. Addressing what is considered a good enough performance early on during an implementation project may highlight the different perspectives of all involved, but it is unlikely to be sufficient. Ongoing communication, reassessment and adjustment of these metrics is critical to ensure meaningful alignment, as a one-off agreement can lead to misaligned expectations and inconsistent model performance across regulatory authorities.

### 3.7 Transparency, explainability and governance

While federated learning offers significant advantages for privacy-preserving AI system development, regulatory authorities must establish clear guidelines for its use and for interpreting its outcomes. This is particularly crucial in a regulatory context where regulators might not fully understand the advantages and disadvantages of federated learning, especially regarding the quality of model outputs.

A sufficient level of explainability is necessary not only to act upon a model’s decision but also to comprehend why that decision was made. Ideally, in a regulatory framework, the model’s decision should align with what an experienced regulator would decide in similar circumstances. Even if the outcomes differ, a transparent model that incorporates mechanisms for explaining decisions—despite operating within a decentralized framework—can provide regulatory authorities with the insights needed to evaluate and respond appropriately to model outcomes. This approach aligns with strategies observed in multi-database studies, where local analyses allow participating entities to maintain control over their data and perform analyses within their own environments, thereby enhancing transparency and trust in collaborative research efforts (Gini et al., 2020).

One method to achieve explainability in federated learning involves each participating agencies generating feature importance scores independently. For instance, regulatory agencies working together on a federated model to identify high-risk medical devices could independently evaluate which device characteristics, such as product type or previous incident history, significantly influence the model’s risk predictions on their own data. These local insights, when summarised without revealing sensitive or proprietary information, can then be securely shared and combined, offering regulators a comprehensive yet privacy-safe understanding of critical risk factors across the entire federation. Importantly, federated learning does not remove the responsibility of individual agencies to perform rigorous local explainability analyses, which remains an essential task at the local level.

## 4 TRICIA: a use case for federated learning in risk assessment

Based on the previous section, we believe that federated learning is a promising solution to some of the challenges that regulatory science faces in data-centric collaboration. To demonstrate this, we present the federated learning use case TRICIA as a proof of concept.

### 4.1 Background

The global medical devices market size was valued at USD 518.46 billion in 2023 and is projected to grow from USD 542.21 billion in 2024 to USD 886.80 billion by 2032 (Medical Devices Market Share, 2025). This development has a direct impact on regulatory authorities. The Swiss legislation stipulates two mandatory routes in the reporting process for serious incidents involving medical devices (MedDO, 2020; IvDO, 2022). Any professional who becomes aware of a serious incident when using medical devices must report it to both the supplier and Swissmedic. Likewise, the manufacturer of a device made available in Switzerland must report all serious incidents to Swissmedic. If all parties comply with their obligations, Swissmedic receives two reports of the same serious incident: one from the user and one from the manufacturer. This ensures that Swissmedic is informed about every serious incident. Similar legislation exists in other countries and the information to be reported is, at least in Europe, very similar across jurisdictions.

In 2023, Swissmedic received around 5,500 reported serious incidents, which corresponds to an increase of 5.4% compared to the previous year. The incoming reports are processed by a scientific officer of the medical device vigilance department in a triage process for later case handling. In this process, a risk score is assigned per case according to an internal risk assessment process. The cases are then handled on a risk-basis.

A part of the triage process was automated to assist the scientific officers using a decision support system that assesses three aspects of risk. This risk assessment includes: the harm that occurred (severity), the number of serious incidents that have occurred with the device in each period (probability) and whether an issue of the medical device can be detected before a harmful effect occurs (detectability). Using transformer ML models, an accuracy of 81.44% for severity, 81.16% for detectability, and 92.82% for probability was achieved. Based on these results and an extensive testing phase, the medical device vigilance department decided to integrate the tool into their standard operating procedures.

The tool has been presented at various conferences such as the Global Coalition of Regulatory Sciences 2022 and has aroused the interest of other regulatory authorities that are also interested in tools to support their risk assessment. However, these regulatory authorities cannot use the tool in its current form as the underlying models are specific to Swissmedic as they were trained on reports submitted from Switzerland. To increase the generalisability of the models, they would have to be retrained or adapted using data from other regulatory authorities. Furthermore, and as mentioned above, regulatory processes within regulatory authorities may vary so that the specific predictions they seek to make vary requiring additional complexity in model fitting.

#### 4.1.1 Objective

By using federated learning, the existing TRICIA models are to be continuously improved with new data from our partner regulatory authorities without the need for direct access to their data.

#### 4.1.2 Data preparation

First, for each regulatory authority (i.e., for each client), the serious incident reports must be extracted from the production system. Augmenting real data with synthetic data has been demonstrated to enhance the performance of federated learning models (Using Foundation Models, 2024). Synthetic data can be used to compensate for underrepresented risk classes, further improving the generalisability and convergence of the model. In such a scenario, synthetic data would be combined with real data to make the model more robust and versatile. Finally, data must be protected from training by differential privacy measures.

#### 4.1.3 Round of federal learning

Once the volume of new data reaches a certain threshold, a new round of federated learning can be started. First, the global model with the latest version of the weights is sent to each agency. Each authority trains the model with the new data and creates a local version of the model. These local versions are then sent back to the central server, which merges the weights and creates a new global version of the model. This updated version can then be sent back to all regulatory agencies for testing and use. It is expected that after several rounds of learning, the model will continue to improve its ability to classify reports by severity. Finally, the model will be used to trigger alerts on new incoming reports in the production system and make risk assessment more efficient.

### 4.2 Proof of concept implementation

**FIGURE 2:**
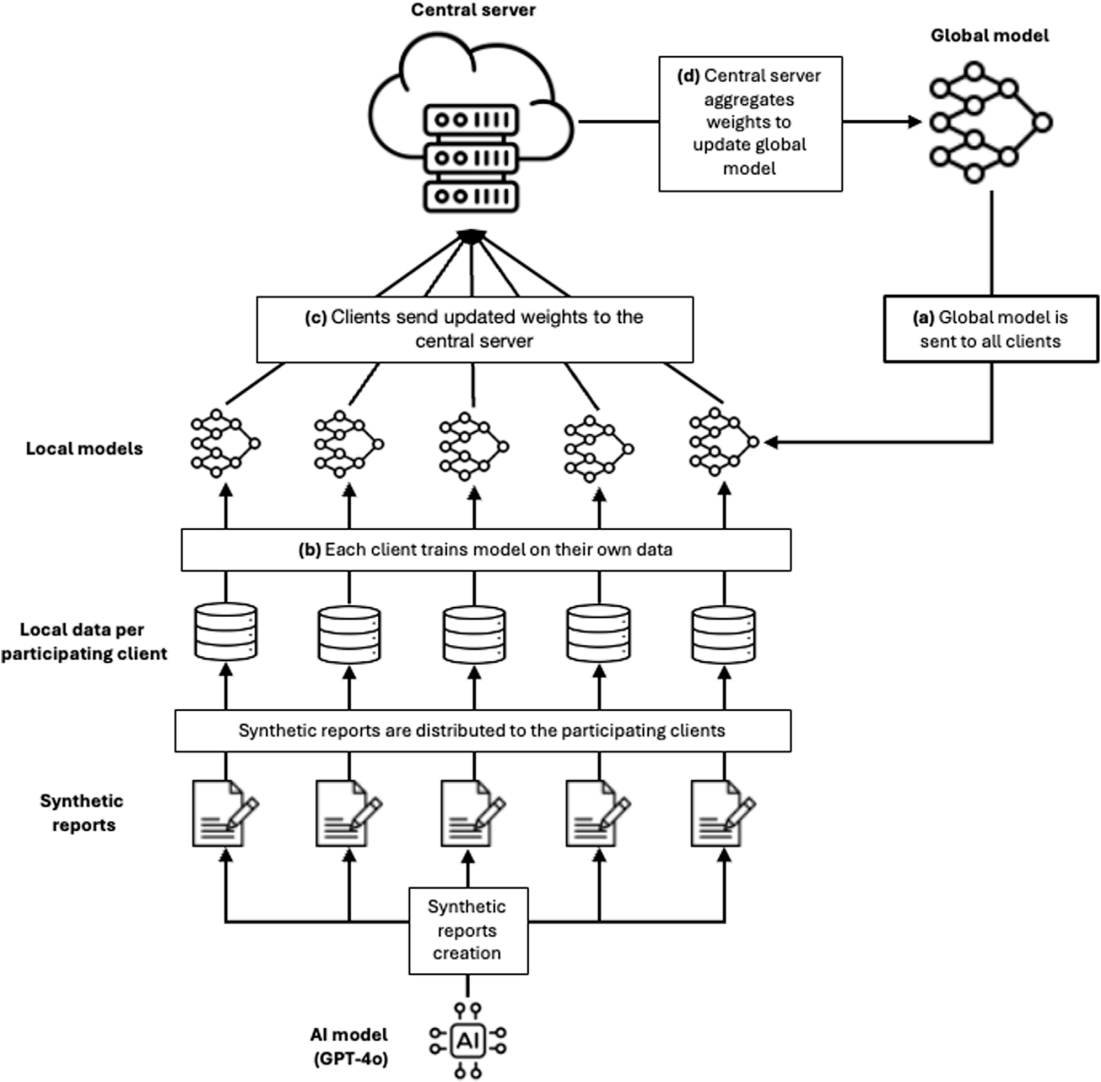
Pipeline developed as a proof-of-concept in TRICIA federated learning. Five clients are participating in the round of learning. All clients use synthetic data generated by OpenAI’s’ GPT-4o model. In addition to the federated learning process (described in Figure 1), the regulatory agencies are represented as clients. During the process, regulatory agencies are responsible for aggregating their data, augment them with synthetic data and add differential privacy layer to improve security.

At the 2024 Global Summit on Regulatory Sciences in Little Rock, USA, several working groups collaboratively piloted innovative technologies to assess their potential applications in regulatory science. One of the working groups focused on validating the TRICIA use case as a proof of concept by deploying and testing an end-to-end TRICIA federated learning pipeline. This pilot involved five participating clients from Swissmedic, the US Food and Drug Administration (FDA) and the Danish Medicines Agency (DKMA) (see Figure 2). The objectives were to gain practical experience, demonstrate the added value of federated learning in regulatory science, and identify potential implementation pathways along with expanding the use case of similar federated learning pipelines.

For demonstration and reproducibility purposes, we did not use any sensitive data but used OpenAI’s GPT-4o model to generate five synthetic datasets, each representing incident reports related to a specific type of medical device, such as wearable medical devices, software as a medical device, and different types of implants. Each dataset contained 500 entries with class labels representing the severity, maintaining a distribution consistent with natural occurrence and following a similar few-shot prompting template^1^. A validation set of 100 entries was randomly selected from these synthetic datasets to ensure equal representation across the datasets.

The federated learning experiment was conducted using a deep learning model (BiomedNLP-PubMedBERT) over three training rounds. Each round comprised five epochs and employed an 80/20 random train/test split performed on each independent client. Individual clients, representing different medical device types, demonstrated low predictive performance, with the highest accuracy being 41.31% for the “Software as Medical Device” client. However, the joint federated model outperformed individual clients, achieving an overall accuracy of 49.01%. Despite limitations such as the lack of fine-tuning, the synthetic nature of the data and the small sample size, these results highlight the potential of federated learning to improve performance compared to standalone solutions. The performance of the proof-of-concept federated model appears modest, and several factors contributed to these results. The limited dataset size was a key factor, as each client had only 500 synthetically generated incident reports, resulting in a total of less than 2,500 samples to train our classification model, whereas this number should typically be several times higher. This small, artificial dataset likely resulted in underfitting and high variance, as it may not capture the nuanced patterns and noise of real-world data. In addition, the minimal training of the pilot study - only three rounds with five local epochs per client and without extensive hyperparameter tuning - likely underutilized some of the model’s capacity. The inherently challenging task of predicting event severity from free-text reports, potentially complicated by unbalanced multi-class data and data heterogeneity across device types, also contributed to the modest performance. Importantly, our primary objective was not to achieve peak performance *per se*, but to demonstrate that a shared global model can deliver measurable improvements over isolated local models, thereby validating the potential of federated learning.

### 4.3 Considerations and lessons learned

Here some key implementation considerations and lessons learned are thoroughly discussed and summarized below:

**Heterogeneity of the data:** In addition to standardised data classification, other data requirements must be met to ensure the smooth functioning of the federated learning pipeline. All participating organisations must maintain standardised data structures and characteristics. In this application, the input data is text data, which has the advantage that no features need to be created or edited. However, text data may differ from one authority to another; for example, one authority may submit reports with a significantly higher average word count or use different terminology. In addition, different processes within these agencies may result in different predictive labels or different definitions of risk, meaning that the target variables may not be consistent across clients. This can add complexity to model training as the model has to account for these differences in predictive targets and risk assessments. Although these differences can improve the generalisability of the model and do not necessarily need to be standardised, quantifying these differences could provide valuable insights into the performance of the model. Such analysis could help to assess the adaptability of the model and understand how it responds to different textual data, which could ultimately help to optimise the federated learning pipeline.

**Homogeneity of the data:** Although the textual data may differ between regulatory authorities, for certain use cases and in specific economic markets, the same reports are often used in a structured data format. Much of this data can be considered homogeneous and could serve as an excellent candidate for federated learning. A TRICIA-like use case, which automatically assesses incident reports, could be expanded to several markets, including the European Union, where the incident report format is standardized. Since this form is harmonized across all EU member states, a federated learning approach can particularly benefit smaller countries with limited data. For example, countries that receive fewer than a specified number of incidents annually or those lacking robust databases for storing national incident data related to medical devices could leverage federated learning to improve analysis and decision-making. While markets outside the EU may have different reporting criteria, most regulatory authorities in fields such as medical devices already use, or aim to use, structured coding to describe incidents. Another potential use case arises where different markets utilize the same structured information to make varied decisions. Federated learning could facilitate a harmonized approach to assessment or, at the very least, support the alignment of decision-making processes across these markets. Although there are several benefits, proper consideration of the impact of both the heterogeneity and homogeneity of the data is crucial when evaluating whether federated learning is the optimal choice for a regulatory authority’s desired use case.

**Number of clients:** Since the clients of federated learning in our feasibility are regulatory authorities, we work in a cross-silo^2^ configuration. Unlike cross-device configurations, which involve many individual devices, in cross-silo federated learning a small number of organisations work together to train models. These participants are usually organisations with larger data sets and a long-term interest in collaboration. However, cross-silo federated learning comes with specific challenges, including incentivising participation, ensuring security, confidentiality and privacy, and optimising performance and scalability. As the number of participating regulatory authorities is likely to be small, especially in the initial phase, the absence of one or more regulatory authorities in a learning round could significantly affect the performance and further development of the model. In extreme cases, a regulatory authority could repeatedly withdraw from the learning rounds and still benefit from the global model that has been trained by the others. To avoid such scenarios, trust between the participating organisations is crucial, as it is not possible to track exactly which clients are participating in each learning round. This trust forms the basis for a fair and co-operative environment in which all participants actively contribute to improving the overall model and jointly benefit from the progress made.

**Computational workload:** As a BERT-based model with many parameters, the TRICIA model requires considerable computing resources for training. The regulatory authorities participating in the federated learning process must therefore have sufficiently high computing capacities, including access to GPUs. This requirement poses a particular challenge as it could prevent some regulatory authorities from participating in the process, especially if they do not have the necessary infrastructure or the expertise required for the effective use of AI/ML models within a federated learning context. This could affect the diversity of data sources and the quality of the global model. It is therefore important to develop strategies that enable less well-equipped regulatory authorities to participate in the federated learning process, e.g., by utilising cloud services.

**Alignment on downstream model trainings:** Ultimately, the regulatory authorities must establish a consensus regarding the subsequent course of action following the conclusion of the preliminary training phase. This entails determining whether the global model should be maintained in its current, static form or whether it should undergo periodic updates to reflect new insights gained from other clients over time. It is vital that regulatory agencies establish transparent and consistent protocols regarding the frequency, governance, and criteria for updates. This will ensure that the models perform consistently while minimising the risks of data drift or bias. It is of the utmost importance that alignment is achieved across agencies on these standards to foster trust and interoperability in the use of federated learning for regulatory purposes.

## 5 Conclusion

To summarise, federated learning is a promising way for regulatory authorities to improve collaboration while preserving data privacy. As the focus is on sharing model weights rather than raw data, privacy concerns are inherently addressed in federated learning. However, the successful integration of this approach into the regulatory framework, as illustrated in the TRICIA use case, requires careful consideration of legal, ethical and security aspects. To ensure the integrity of this innovative approach, accountability, compliance and secure communication channels must be ensured.

To realise the full potential of federated learning, it is critical that regulatory authorities not only collaborate on technical implementation but also identify common use cases based on shared interests and data sources. Especially in use cases where the core task is considered similar and reporting criteria are identical to those in the EU market, federated learning can greatly benefit a harmonized assessment procedure and enhance decision-making through technology. Importantly, the effectiveness of federated learning increases with the number of participating clients, further emphasising the importance of joint efforts. The proof of concept conducted at GSRS24 successfully demonstrated the feasibility of the TRICIA federated pipeline, setting the stage for future collaborations between regulatory authorities. However, the full potential of this work will be realized as more authorities participate in the project and the pipeline is widely deployed.

## Data Availability

The original contributions presented in the study are included in the article/supplementary material, further inquiries can be directed to the corresponding author.

## Footnotes

1 In our case, few-shot prompting means that for each medical device, a few example cases were created by hand and shown to the GPT-4o model to aid its generation of synthetic incident reports.

2 Cross-silo means a small number of highly trusted contributors participate in the training process, each providing their own data as training source.
